# Diabetic Cardiomyopathy Modelling Using Induced Pluripotent Stem Cell Derived Cardiomyocytes: Recent Advances and Emerging Models

**DOI:** 10.1007/s12015-018-9858-1

**Published:** 2018-10-20

**Authors:** Cecilia Granéli, Ryan Hicks, Gabriella Brolén, Jane Synnergren, Peter Sartipy

**Affiliations:** 1 Systems Biology Research Center, School of Bioscience, University of Skövde, SE-541 28 Skövde, Sweden; 2 Discovery Sciences, IMED Biotech Unit, AstraZeneca Gothenburg, SE-431 50 Mölndal, Sweden; 3 Global Medicines Development, CVRM, AstraZeneca Gothenburg, SE-431 50 Mölndal, Sweden

**Keywords:** Induced pluripotent stem cells, Cardiomyocytes, Diabetic cardiomyopathy, Disease modeling, Insulin resistance

## Abstract

The global burden of diabetes has drastically increased over the past decades and in 2017 approximately 4 million deaths were caused by diabetes and cardiovascular complications. Diabetic cardiomyopathy is a common complication of diabetes with early manifestations of diastolic dysfunction and left ventricular hypertrophy with subsequent progression to systolic dysfunction and ultimately heart failure. An in vitro model accurately recapitulating key processes of diabetic cardiomyopathy would provide a useful tool for investigations of underlying disease mechanisms to further our understanding of the disease and thereby potentially advance treatment strategies for patients. With their proliferative capacity and differentiation potential, human induced pluripotent stem cells (iPSCs) represent an appealing cell source for such a model system and cardiomyocytes derived from induced pluripotent stem cells have been used to establish other cardiovascular related disease models. Here we review recently made advances and discuss challenges still to be overcome with regard to diabetic cardiomyopathy models, with a special focus on iPSC-based systems. Recent publications as well as preliminary data presented here demonstrate the feasibility of generating cardiomyocytes with a diabetic phenotype, displaying insulin resistance, impaired calcium handling and hypertrophy. However, capturing the full metabolic- and functional phenotype of the diabetic cardiomyocyte remains to be accomplished.

**Electronic supplementary material:**

The online version of this article (10.1007/s12015-018-9858-1) contains supplementary material, which is available to authorized users.

## Introduction

The prevalence of diabetes has drastically increased over the past decades from around 110 million in 1980 to over 420 million patients globally in 2017. With approximately 4 million deaths caused by diabetes during 2017 the disease not only imparts huge sufferings amongst patients and their families but is also an enormous burden on health care systems worldwide [1, 2]. Recent predictions are estimating that around 630 million people (i.e. around 10% of the adult population) will suffer from this chronic disease in 2045. Diabetes is expected to increase most in regions undergoing economic development, where the economy is transitioning from low-income to middle-income levels with subsequent changes in lifestyles [1]. Importantly, over 90% of all diabetes cases are type 2 diabetes mellitus (T2DM). Although there are both genetic and epigenetic factors increasing the susceptibility of an individual for the disease, the major risk factors underlying the global increase in T2DM prevalence include obesity, sedentary lifestyle, and poor diets [3–5].

Patients with T2DM also commonly suffer from complications and co-morbidities with around 50% of patients presenting with microvascular complications such as diabetic nephropathy, neuropathy, and retinopathy and around 30% suffer from macrovascular complications such as cardiovascular disease [6]. Among patients with T2DM, cardiovascular complications are the leading cause of morbidity and mortality [7]. This common denominator includes conditions such as coronary artery disease (CAD), myocardial infarction, hypertension and peripheral vascular disease. In general, the rates of mortality due to heart disease and stroke for adult patients with T2DM are around two to three times higher than for those without diabetes [8, 9]. However, the actual risk for cardiovascular complications varies across geographical regions with differences in income levels and ethnicity. Interestingly, the incidence of cardiovascular complications in patients with T2DM has drastically decreased in recent years for patients in developed countries. For example, the risk of a T2DM patient in the US suffering a myocardial infarction or stroke has decreased by almost 70 and 50%, respectively over the last two decades [10]. Improvements in cardiovascular outcomes have also been observed in large clinical trials with sodium and glucose co-transporter 2 (SGLT2) inhibitors developed for the treatment of T2DM [11, 12]. Nevertheless, there is still a major unmet medical need and even with well managed blood glucose levels, patients diagnosed with T2DM still have an increased risk of developing CAD and suffering a stroke as compared to people without diabetes [13, 14].

Diabetic cardiomyopathy (DCM) is a common long-term complication among T2DM patients. The condition is defined as diabetes-associated structural and functional abnormalities of the myocardium, not directly attributable to other confounding factors such as CAD or hypertension. More specifically, the early stages of DCM, although commonly asymptomatic, entails left ventricular hypertrophy, resulting in abnormal left ventricle filling and diastolic dysfunction. As the disease progresses, DCM can lead to systolic dysfunction and heart failure [15]. Although DCM is now recognized as a serious complication of T2DM, the lack of distinct diagnostic criteria has resulted in highly varying reports regarding prevalence, from 16% in the Strong heart-study to other reports with over 60% prevalence [16–21]. In an attempt to unify the clinical evaluation, a four-stage classification of DCM with clear diagnostic criteria has been proposed [22, 23]. Presently, echocardiography is the standard clinical diagnostic tool for DCM. However newer techniques including magnetic resonance imaging, radionuclide scans or positron emissions tomography imaging are emerging as diagnostic alternatives. In addition, quantification of plasma biomarkers such as cardiotrophin-1, activin A, heart-type fatty acid binding protein and insulin-like growth factor-binding protein 7 may be complementary disgnostic tools and give further information related to cardiac hypertrophy, contractile dysfunction, steatosis, and insulin resistance [24, 25].

Although there have been many years of research on the subject, the underlying pathophysiologic mechanisms of DCM are still not completely mapped out, mainly due to the complex network of factors involved in the development of the condition. In addition to clinical research and in vivo studies using animal models, it is also of great importance to develop in vitro models that can accurately recapitulate several of the key processes of the diabetic heart, to further elucidate the mechanisms involved. The cell source is a key factor for the establishment of any in vitro model. Primary human cardiomyocytes (CMs) are difficult to obtain and isolate and the rodent equivalents may lack translatability to the human physiology. However, CMs derived from human induced pluripotent stem cells (iPSC) may represent a viable alternative [26]. These cells are also amendable for genetic modification and as such may provide additional possibilities for disease modelling in a dish [27, 28].

In this review, we will present a summary of the important molecular mechanisms involved in the development of DCM. We will cover aspects of insulin resistance, substrate utilization and metabolism, oxidative stress, mitochondrial function and Ca^2+^-handling and discuss how these processes connect to the pathophysiology of DCM. However, the focus is mainly on how these factors relate to a prospective iPSC-based disease model. Currently existing in vitro models, their strengths and limitation will also be discussed. Finally, we will highlight some recent data from our lab related to the development of insulin resistance in human iPSC-derived CMs in an emerging DCM model.

## Molecular Mechanisms of DCM

Due to the constant workload executed by the heart, it is highly dependent on a continuous generation of ATP to meet the energy demand. In the healthy heart, around 70% of the ATP is generated by lipid oxidation and the remaining 30% of the energy supply comes mainly from glucose metabolism [29, 30]. However, CMs are “substrate-promiscuous” and have the ability to also use lactate, amino acids and ketone bodies for generation of ATP [31]. In comparison to glucose oxidation, the oxidation of fatty acids (FA) generates more ATP per molecule, but at higher rates of oxygen consumption. Under anaerobe conditions (e.g., during acute ischemia), it is therefore more favorable to use glucose as a source of energy compared to FA [32]. In fact, one key feature of the healthy heart is the ability to temporarily adapt to different conditions in oxygen availability and workload by utilizing different substrates, exercising its metabolic flexibility [33].

Even though the oxidation of FA is central in cardiac energetics, glucose metabolism is also important in maintaining functionality in the healthy heart. For example, during exercise, the relative amount of energy produced by glucose oxidation in the healthy heart is increased [32]. Glucose is actively taken up by CMs through insulin dependent glucose transporter GLUT4. As insulin binds the insulin receptor (IR), downstream signaling leads to translocation of GLUT4 to the cell surface and increases uptake of glucose [34]. There is a reciprocal relationship between FA and glucose uptake and metabolism in which high levels of circulating FA triggers an increased expression of PPAR-alpha leading to, not only increased FA uptake and oxidation, but also the inhibition of glucose oxidation. Similarly, oxidation of glucose results in inhibition of CPT-1, the rate-limiting enzyme for mitochondrial uptake of FA [32].

**Fig. 1:**
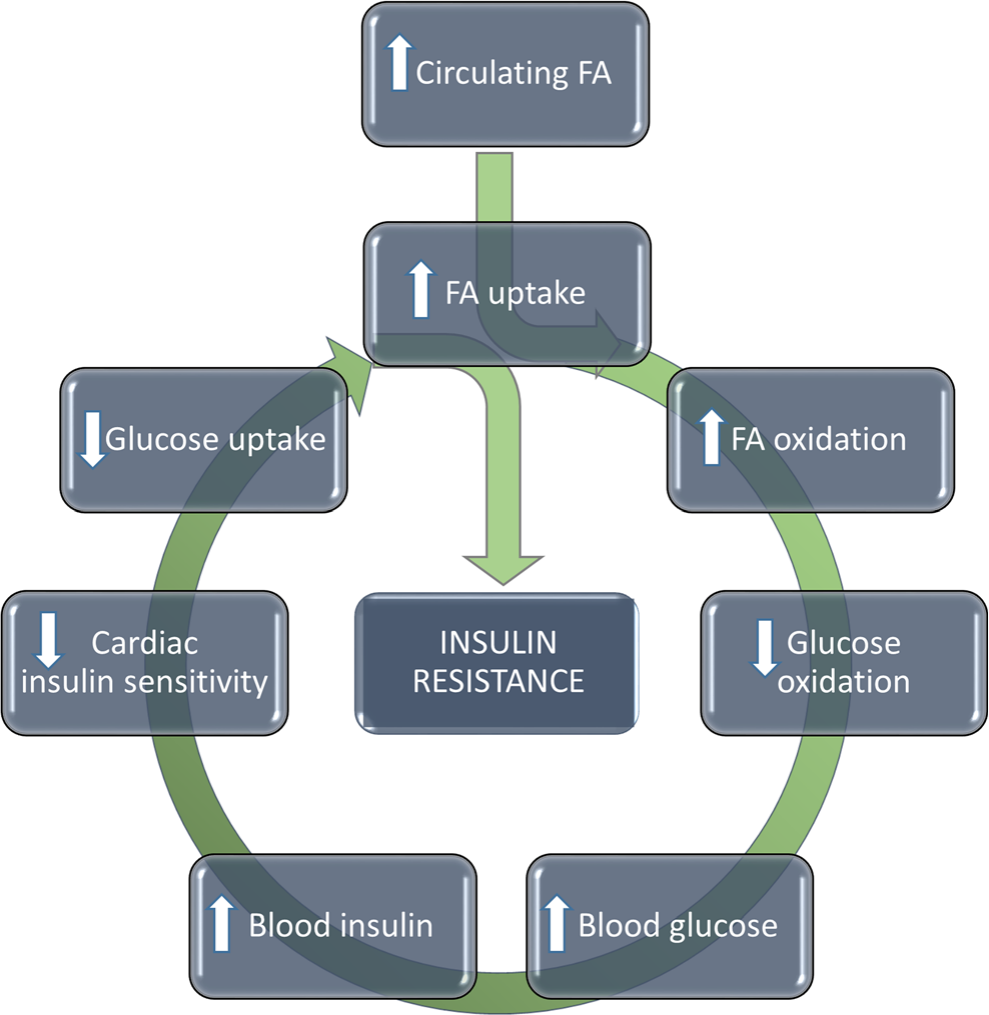
**Development of cardiac insulin resistance** Increased levels of circulating FA will trigger an increase in FA uptake and oxidation, which in turn will suppress glucose oxidation. To counteract rising blood glucose levels, more insulin will be secreted by the pancreas resulting in increasing blood insulin levels. As the cardiac insulin sensitivity is reduced, glucose uptake is further diminished and cells will become further reliant on FA for energy metabolism. As this feedback loop continues the resulting negative spiral will ultimately result in a state of insulin resistance and metabolic rigidity

The onset of insulin resistance in the diabetic heart is commonly attributed to obesity and diets with high fat content resulting in increasing levels of circulating FAs. However, although these factors are central triggers initiating the development of cardiac insulin resistance, there are several other pathophysiological processes contributing to impaired myocardial insulin sensitivity. These processes have been thoroughly reviewed elsewhere [35] and here we will present a generalized summary of the main mechanisms leading to cardiac insulin resistance, schematically illustrated in Fig. 1.

With increasing circulating FA delivered to the pre-diabetic heart, there is an increased FA uptake and oxidation in the tissue [36]. As a consequence, glucose oxidation is suppressed and glucose uptake is diminished. In parallel, a similar process will result in that also the skeletal muscle tissue will have reduced glucose uptake and thus the blood glucose level rises (hyperglycemia). Higher blood glucose levels are initially compensated by increased pancreatic insulin secretion (hyperinsulinemia) leading to decreased myocardial insulin sensitivity which then further decreases glucose uptake and oxidation [37–40]. This negative spiral of substrate utilization feedback will ultimately lead to CM insulin resistance. With insulin resistance, the increased reliance of FA oxidation for ATP generation is reinforced and the cells suffer from a state of metabolic rigidity.

As described above, the metabolic shift in the diabetic CMs due to insulin resistance and increased circulating FA leads to an increased cellular FA uptake. Circulating FA are mainly taken up into the cell by the FA transporter CD36. The FA are converted, intracellularly, to fatty acyl-CoA, which subsequently either is transported to the mitochondria for oxidation, or further converted to triacylglycerol for intracellular storage [41]. Although the diabetic state entails increased FA oxidation, there is also an increase in FA conversion to triglycerides. This lack of balance between uptake, usage, and storage of FA are characteristic of the diabetic heart and leads to excessive intracellular lipid accumulation [42, 43].

There are several proposed mechanisms by which the altered substrate availability and utilization associated with T2DM may result in cardiac dysfunction, and many of the processes appear highly interconnected. Increased FA oxidation has been demonstrated to lead to increased intracellular reactive oxygen species (ROS) generation, possibly through an increased electron transport chain flux [44, 45]. It has been hypothesized that this may upregulate mitochondrial uncoupling protein activity and increase proton leakage to reduce the mitochondrial membrane potential and thereby the amount of ROS produced [45, 46]. However, increased uncoupling will lead to increased cardiac oxygen consumption without equivalent increase in ATP generation, resulting in a net reduction in cardiac efficiency [47, 48]. The strain on the diabetic heart with regard to cardiac efficiency is thereby dual, with decreased efficiency due to increased reliance on FA oxidation (requiring more oxygen) as well as increased mitochondrial uncoupling.

The increased intracellular lipid pool of the diabetic CM is thought to be a factor with several detrimental effects, commonly referred to as lipotoxicity. However, it is likely that these effects are not directly attributed to the actual lipid pool but instead to toxic lipid intermediate metabolites [41]. Intracellular lipid accumulation also makes the cell more susceptible to lipid peroxidation by ROS, and lipid membrane damages as a consequence [49]. Several studies in animal models have revealed that not only ROS but also advanced glycosylated end-products (AGEs) generated under lipotoxic and/or hyperglycemic conditions can cause cardiac fibrosis, hypertrophy and stiffness. The AGEs have been implicated as links between altered cardiac metabolism and the development of contractile dysfunction in the diabetic heart [43, 50–52].

Another proposed link between the altered cardiac metabolism, characteristic for the diabetic heart, and functional impairment, such as diastolic and systolic dysfunction, is the intracellular Ca^2+^-handling. CMs isolated from diabetic [db/db] mice displayed both impaired Ca^2+^ transients and reduced diastolic and systolic Ca^2+^ levels. There was decreased expression of the main cardiac Ca^2+^ transporter SERCA2a and increased Ca^2+^ leakage resulting in reduced Ca^2+^ flux potential [53, 54]. Furthermore, it has also been demonstrated that SERCA2a is impaired by increased levels of myocardial AGE in a transgenic mouse model [55]. The resulting reduced trans-membrane calcium gradient and impaired Ca^2+^ flux potential may cause altered resting potential, which manifests as diastolic dysfunction.

Since the heart is highly dependent on mitochondrial energy metabolism to ensure continuous functionality, mitochondrial dysfunction has been proposed to have a key role in the progression of diabetic cardiac disease. Mitochondrial functions, which are impaired or dysregulated in the diabetic heart, do not only include ROS generation and uncoupling of the electron transport chain (ETC) as discussed above, but also mitochondrial fusion, fission and mitophagy [56].

## In Vitro Disease Models of DCM

There are several animal models that have been developed to study the effects of diabetes and its complications. Examples include the genetically compromised [ob/ob] and [db/db] mice but diet induced diabetes models, which manifest cardiac complications, are also commonly used [57, 58]. However, for further molecular insights, cell based models are valuable tools and can be used to study the underlying mechanisms of DCM in more detail. At present, there are several established disease models based on iPSC-CMs for diseases with genetic cause, especially monogenic disorders [59]. However, with the multifactorial, lifestyle-related etiology of DCM, disease modelling becomes more complex. In addition to generating further mechanistic insights into disease progression, a clinically relevant in vitro DCM-model would be a great resource in pre-clinical drug discovery and development both for target identification and candidate drug validation.

**Fig. 2:**
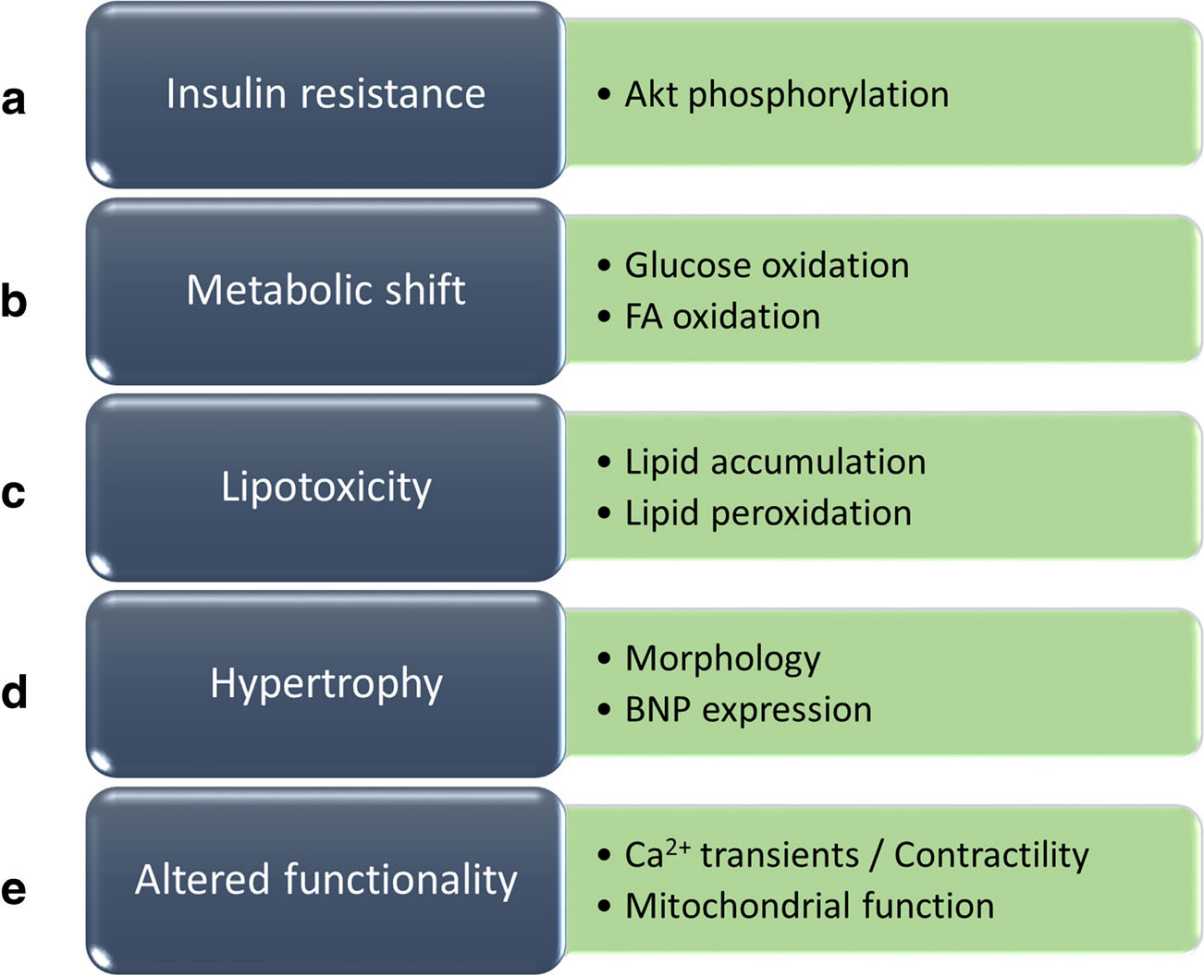
**Key DCM model requirements including relevant readouts** (**a**) Insulin resistance – demonstrated by reduced insulin signaling, for example reduced Akt phosphorylation. (**b**) Metabolic shift – demonstrated by reduced glucose oxidation and increased FA oxidation. (**c**) Lipotoxicity – demonstrated by increased intracellular lipid accumulation and/or increased lipid peroxidation. (**d**) Hypertrophy – demonstrated by hypertrophic morphology and/or increased expression of hypertrophic markers such as BNP. (E) Altered functionality – demonstrated by impaired Ca^2+^-transient or contractility and/or mitochondrial dysfunction

In order to model DCM in vitro we propose that the following minimal requirements should be met by the model: Insulin resistance, metabolic shift, lipotoxicity, hypertrophy and altered functionality (Fig. 2). To date and to our knowledge, there is yet no published model that fulfills all of these requirements simultaneously. However, Drawnel and colleagues proposed an in vitro model of DCM based on iPSC-derived CMs, which did fulfill a majority of these criteria [60]. In addition, the model was implemented in a small molecule screen identifying drugs that preserved a healthy cardiomyocyte phenotype in vitro. The model is based on the induction of a diabetic phenotype by subjecting the cells to an extracellular milieu reminiscent of the diabetic state. Firstly, the cells were kept in a glucose free, FA containing medium and the authors hypothesized that the cells would undergo a metabolic shift in the absence of glucose and alter their metabolism to mainly oxidize FA as a source for energy. Thereafter, a culture medium was introduced consisting of intermediate glucose levels, endothelin-1 and cortisol. The authors performed extensive model characterization and presented a diabetic CM-phenotype with increased lipid accumulation, oxidative stress, increased release of the hypertrophy marker BNP and altered Ca^2+^ transient. However, an important aspect lacking in the characterization of the model was how the diabetic milieu affected the insulin sensitivity and metabolism in the cells. This would be of key interest for studying the pathophysiological processes of DCM connected to for example diabetic CM substrate utilization.

Since the decreased insulin sensitivity/insulin resistance is central for the progression of T2DM and its cardiovascular related complication, including DCM, induction of insulin resistance is highly desirable. Recently, insulin resistance has been achieved in both human iPSC- and human embryonic stem cell (hESC) derived CMs by lipid overload. In culture medium containing high levels of palmitate, the cells displayed reduced insulin signaling (Akt phosphorylation) and glucose uptake [61, 62]. Therefore, it seems likely that lipid overload, by for example high palmitate exposure, could be a promising strategy in establishing a metabolically relevant iPSC-based DCM model. However, since palmitate also is known to induce apoptosis in CM, a careful titration of its concentration would be necessary.

In primary rat CMs, insulin resistance has been induced both through exposure to high levels of palmitate but also using uric acid [62–64]. Similarly to the effects on stem cell derived CMs, this leads to reduced insulin signaling and glucose uptake. However, in two insulin resistance models based on primary rat CM, lipid overload has also been demonstrated to generate functional alterations which may have implications in DCM, such as contractile dysfunction [62, 64]. The same effect on contractility was also achieved by repressing Akt1 and Akt2 expression in rat neonatal CMs [65]. High uric acid-induced insulin resistance could be reversed by an antioxidant, indicating a mechanism of insulin resistance through oxidative stress [63]. Although highly relevant, the effects of insulin resistance observed in primary CM in the examples described above have not been investigated in a human model. Therefore, it remains to be determined if a human iPSC-derived model of DCM based on insulin resistance induced by for example lipid overload would recapitulate the diabetic phenotype with regard to substrate utilization and functionality.

Human iPSCs derived from patients with a genetic mutation to the  IR, resulting in severe insulin resistance, have been investigated in comparison to iPSCs generated from healthy individuals. Insulin resistant cells displayed impaired oxidative function, increased susceptibility to oxidative stress and suffered from ATP-depletion [66]. The insulin resistant cells also had increased mitochondrial number and decreased mitochondrial size, suggesting an imbalance in the mitochondrial fusion/fission regulation which has previously been implicated as a key process in the pathophysiology of DCM [56, 67].

In addition to capturing the metabolic phenotype of the diabetic CM, further assessments of the CM functionality as part of a DCM disease model would be of key interest. The diabetic phenotype in iPSC-derived CM described by Drawnel and co-authors displayed impaired CM functionality, demonstrated by reduced Ca^2+^-transient frequency and reduced calcium transient amplitude [60]. In rat CMs, high palmitate induction of insulin resistance did not generate a similar impairment in calcium handling although the cells did display impaired contractility [62].

Two independent studies have investigated the substrate utilization in iPSC-derived CMs, although not from a diabetic perspective, but with regard to CM maturation and resemblance to their in vivo counterpart [68, 69]. Interestingly, it was demonstrated that even though the cells were able to metabolize FA, glucose was their preferred substrate when it was present in the culture medium [69]. This metabolic trait of the iPSC-derived CMs poses an important question with regard to glucose containing medium and substrate dependence, not only from a maturation perspective, but also related to the metabolic shift, characteristic of diabetes. The major finding in the more recent study was however, that the utilization of FA increased in the presence of galactose, and that this supplement led to a more adult like metabolic phenotype. Galactose increased the oxidative capacity and FA oxidation of the cells as well as their contractility, CM-morphology and Ca^2+^-handling whilst also protecting against lipotoxicity and hypertrophy [68]. In combination with high palmitate, galactose would therefore be an attractive medium component in a diabetic context.

**Fig. 3:**
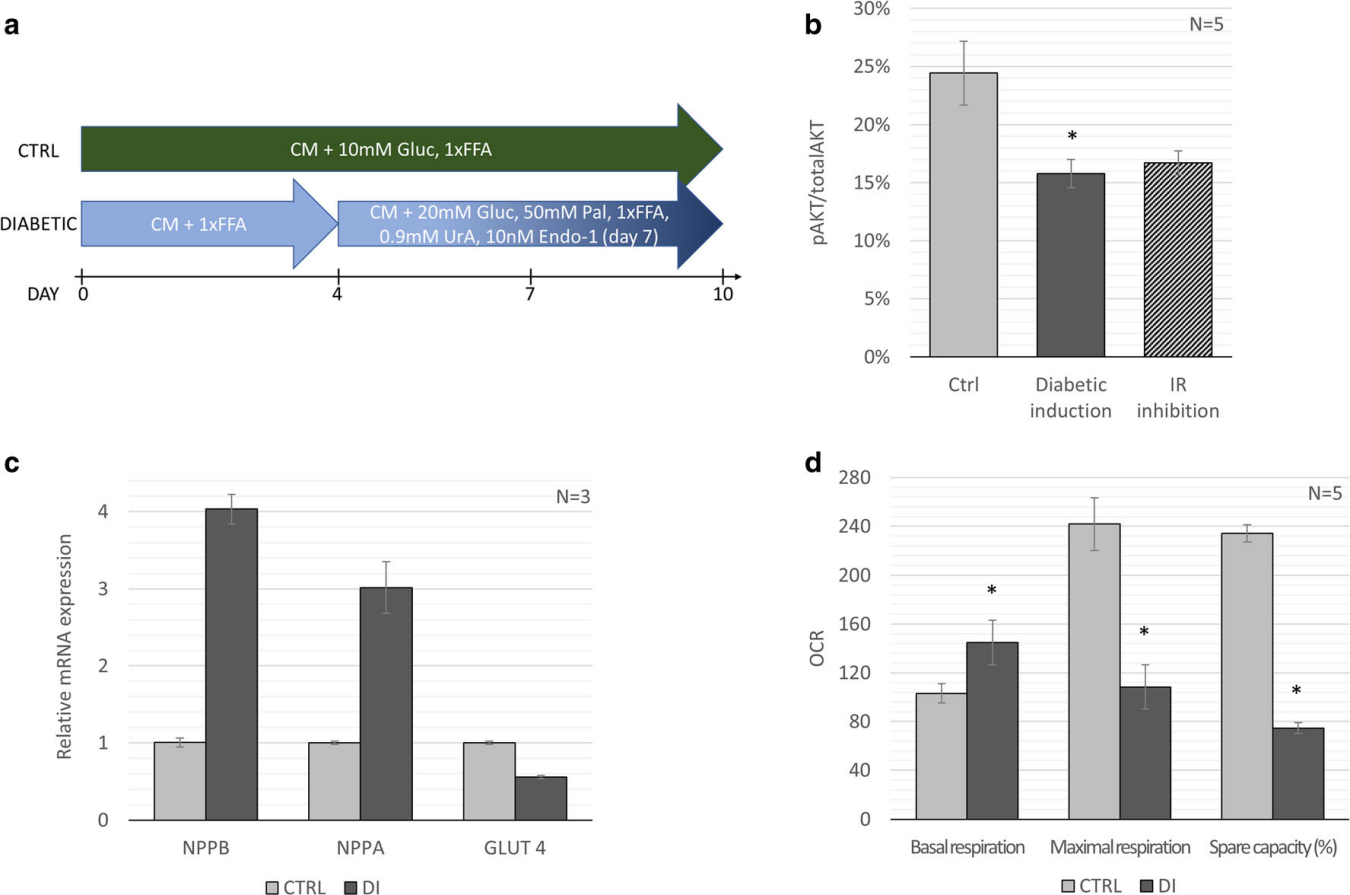
**iPSC derived diabetic cardiomyopathy model** (**a**) Model setup: CMs derived from CDI-MRB iPSCs from Cellular Dynamics International were used in an induction medium protocol. Cardiac maintenance medium (CM): DMEM no glucose, 10mMHEPES, 2mML-carnitine, 5 mM creatine, 5 mM taurine, 1 mM ITS, 1 mM nonessential amino acids, linoleic-oleic acid (1xFFA) supplemented with 10 mM glucose. Maturation medium (MM) consisting of CM supplemented with 1xFFA. Diabetic medium (DM) consisting of CM supplemented with 20 mM glucose, 50uM palmitate (conjugated by 0.8%FAF-BSA), 15 mg/100 ml uric acid, 2xFFA and 10 nM endothelin-1 after day 10. The effect of the diabetic induction protocol on (**b**) Akt(S473)-phosphorylation, (**c**) Relative gene expression of hypertrophy- and substrate utilization markers and (**d**) Cardiomyocyte respiration - oxygen consumption rate measured by Seahorse XF in culture medium before and after oligomycin, 2,4-dinitrophenol and rotenone/antimycin A treatment. Error bars represent standard deviation and * indicates *p* < 0.05 compared to the control by non-parametric two-tailed Mann-Whitney Test

Working towards the establishment of a protocol for modeling DCM in human iPSC-derived CM in vitro, which would fulfil the requirements proposed here, there are still some key issues to be addressed. Inducing insulin resistance in a disease-relevant manner would entail an induction protocol mimicking the extracellular conditions which cells are exposed to in patients with T2DM. Building on previously published protocols for diabetic induction our lab has developed a strategy for induction of insulin resistance by lipid overload (Fig. 3a). By applying this method, substantial reduction in Akt-phosphorylation following insulin stimulation was achieved (Fig. 3b). In order to give context to the effects on Akt phosphorylation, the IR-antagonist S961 was used, which has previously been demonstrated to induce insulin resistance both in vitro and in vivo [70, 71]. In human iPSC-derived CMs subjected to lipid overload Akt(S473)-phosphorylation is decreased to the same extent as the IR-antagonist, indicating a state of poor insulin sensitivity or insulin resistance. These unpublished findings are corroborated by two recent studies in which high FA conditions were demonstrated to reduce Akt phosphorylation and glucose uptake in iPSC-derived CM [62, 72]. Interestingly, in one of these studies, similar results were obtained for induction of insulin resistance by the inflammatory mediator TNF-alpha which could be an alternative strategy for modeling diabetic cardiomyopathy, relating a disease model to the clinical findings of increased presence of pro-inflammatory mediators in patients with T2DM [72, 73].

As a result of the induction protocol from our lab, there was a drastic increase in gene expression of hypertrophy markers NPPB and NPPA (Fig. 3c). Increased expression of NPPB is one of the hallmarks of cardiac hypertrophy and indicates that cells retain a hypertrophic phenotype in the DCM model. In the diabetic heart, the onset of insulin resistance will entail a decrease in glucose uptake. It is therefore interesting to note that also the gene expression of insulin dependent glucose transporter GLUT4 is altered in the model presented here (Fig. 3c). Although the temporal relationship between decreased GLUT4 expression and insulin resistance is unclear, a reduction in GLUT4 expression has been demonstrated in insulin resistant models based on genetically altered iPSCs [66, 74].

Importantly, Burkart and co-authors also established a correlation between insulin resistance (by IR mutation) and altered mitochondrial morphology and function. In their undifferentiated state, insulin resistant iPSC had increased mitochondrial number and decreased mitochondrial size. Furthermore, the mitochondrial oxidative function was also impaired, with decreased spare respiratory capacity [66]. In our model, not only was the spare respiratory capacity reduced, but also the maximal respiration (Fig. 3d). The induction of a diabetic phenotype also results in an increased basal respiration indicating a reduced cardiac efficiency, which is in line with previous finding of DCM in vivo and also in iPSC-derived CMs from Type 1 diabetes mellitus (T1DM) donors [75, 76]. In primary rat CMs, palmitate treatment resulted in decreased ATP levels and maximal respiration [67]. However, in contrast to the present data, lipid overload in the rat based model decreased the basal respiration. A reduction in maximal respiration and spare respiratory capacity could indicate mitochondrial dysfunction, for example an ineffective electron transport chain resulting in ATP depletion. Besides impaired mitochondrial function, altered Ca^2+^ transient or contractility patterns would indicate alterations in functionality connected to a diabetic state in the CM. Although insulin resistance was not established in the model described by Drawnel and co-authors, mimicking the diabetic milieu resulted in decreased frequency of systolic Ca^2+^ transient and amplitude of CM beats [60], capturing functionality impairment seen both clinically in a DCM-patient group and in T2DM-animal models [52, 77].

As mentioned above, one of the unresolved key requirements for an iPSC-based DCM model proposed here is the evidence of a metabolic shift in the cells. Ideally, an in vitro model should capture this increase in FA oxidation and decrease in glucose metabolism. However, with an in vitro model based on iPSC-derived CM, this criterion poses an additional challenge since iPSC-derived CMs are known to be immature with a fetal like phenotype, especially in their metabolic profile, with higher dependency on glucose oxidation [78, 79]. However, much progress with regard to maturation of human iPSC-derived CMs has been made and recently, iPSC-CMs cultured in a 3D environment and subjected to mechanical stimulation were shown to gain properties resembling those of mature CMs with regard to gene expression, ECM-structure, calcium handling and oxidative metabolism [80]. Achieving a metabolically mature CM-phenotype when using iPSC-derived CMs in a disease model is of key importance, especially since DCM is a disease of the adult heart with altered metabolism at the core [81].

## Future Outlook

With the epidemic proportions in the diabetes prevalence, and with cardiovascular disease being the leading cause of mortality and morbidity in subjects with diabetes, research on treatment strategies are urgently needed. In order to further decipher the mechanisms responsible for diabetes related cardiovascular disease, accurate disease models need to be developed that capture the human pathophysiology of diabetic cardiomyopathy. With the availability of such a tool, comes also the possibility to perform screenings of new pharmacological agents and therapeutic strategies. The fast progressing field of iPSC research and more specifically generation of phenotypically and metabolically relevant iPSC-derived CMs offers a promising foundation for disease model development. In contrast to animal based models or models based on primary cells, iPSC is an unlimited source of cells with species-specific relevance to the human physiology, and without the ethical concerns associated with animal experimentation. However, in addition to the specific challenges of iPSC-derived CMs presenting with a fetal-like phenotype/metabolism as discussed above, there are also general challenges with iPSCs related to genomic instability, residual somatic epigenetic memory, and genetic variations. These changes may potentially result in altered differentiation potential and phenotype variation in the cells and thereby limit their utility in disease modelling. Genomic instability represents a key issue when developing clinical applications based on human iPSCs but whether genomic instability would prevent the use of human iPSC for in vitro applications remains to be determined, most likely on a case-to-case basis [82, 83]. Thus, it is important to assess the genomic integrity of the cells continuously during in vitro experimentation, as well as repeating experiments using different iPSC clones, to allow the investigators to determine if any genomic aberrations are causally related to any negative or false impact on the assay results.

This review has been focused on published work of in vitro models of DCM (primarily human iPSC based), and we also present some preliminary data regarding the induction of insulin resistance in iPSC-derived CM. Our results, as well as previously published data from other groups indicate that insulin resistance can be induced by lipid overload in these cells with resulting changes in CM phenotype. However, to fully recapitulate an acquired metabolic syndrome like DCM using iPSC-derived cells additional research remains. It is a challenge to develop a protocol for recreating pathophysiological processes, which in patients develops over several years, in a matter of days or weeks in the culture dish to model a metabolic condition with high accuracy. We believe that future efforts to this end should be focused on the core mechanism of insulin resistance and aspects on how to achieve this in a disease-relevant manner. If successful, such a model would be of great merit for further research on DCM and a powerful tool in identifying novel therapeutic targets or developing treatment strategies for T2DM related heart disease.

## Electronic supplementary material

ESM 1 (DOCX 16 kb)
