# Supplementary material for: Diabetic Cardiomyopathy Modelling Using Induced Pluripotent Stem Cell Derived Cardiomyocytes: Recent Advances and Emerging Models
Source: Stem Cell Rev. 2018 Oct 20;15(1):13–22. doi: 10.1007/s12015-018-9858-1 (PMC6513824; doi:10.1007/s12015-018-9858-1)
Supplement: Supplementary file 1 — (DOCX 16 kb) [file 12015_2018_9858_MOESM1_ESM.docx]

# Supplemental material

iCell cardiomyocytes (Cellular Dynamics International) were used in all experiments. These cells are purified human iPSC-derived CMs and contain a mixture of spontaneously electrically active atrial-, nodal-, and ventricular-like CMs. The cells were thawed and plated at 63k cells/cm^2^ (plating density) in CDI cardiomyocyte plating medium according to the manufacturer’s instructions. For Seahorse analysis, a plating density of 100k cells/cm^2^ was used. All cell culture vessels were pre-coated using 10ng/ml Fibronectin for 1h at 37°C. After 48h, the thawing medium was changed to CDI cardiomyocyte maintenance medium. The cells were cultured in maintenance medium for a minimum of 10 days for stabilization and maturation before initiating subsequent experiments.

Forty-eight hours before initiating experiments, the medium was changed to a cardiac maintenance medium (CM) consisting of: DMEM no glucose, 10mM HEPES, 2mM L-carnitine, 5mM creatine, 5mM taurine, 1mM ITS, 1mM non-essential amino acids and linoleic-oleic acid (1xFFA) supplemented with 10mM glucose. During all experiments, this was used as the control condition. On day 0 of the diabetic induction protocol, the culture medium was changed to maturation medium (MM) consisting of CM without glucose supplementation. On day 4 of the diabetic induction protocol, the culture medium was changed to a diabetic medium (DM) consisting of CM supplemented with 20mM glucose, 50µM palmitate (conjugated by 0.8%FAF-BSA), 15mg/100ml uric acid and 2xFFA. Finally, 10nM endothelin-1 was added on day 7. On day 10 after induction, the cells were analyzed for insulin signaling by Akt phosphorylation, mitochondrial respiration by Seahorse XF, and gene expression by RT-PCR.

For analysis of Akt phosphorylation, the MSD kit for pAkt/total Akt was used (Mesoscale, K15100D-2). The cells were starved for 3h before analysis in medium without insulin and subsequently stimulated with 200nM Insulin for 10min before lysis. A negative control without insulin stimulation was used as reference. The samples were analyzed according to the manufacturer’s instructions.

The Seahorse cartridge was hydrated in XF-buffer overnight according to the manufacturer’s instructions (Agilent Technologies). The injection ports of the cartridge were loaded with Oligomycin, 2,4-dinitrophenol or Rotenone/Antimycin stock solution resulting in a final concentration of 0.8µM, 200µM and 0.8µM respectively. The cells were equilibrated in a non-CO_2_ incubator 1h prior to analysis and the oxygen consumption rate (OCR) was subsequently analyzed using the Seahorse XF96 analyzer. After Seahorse analysis, the cells were stained using Hoerscht and the nuclei counted.

For gene expression analysis, RNA was isolated using RNeasy mini kit (Qiagen) and transcribed to cDNA using a high capacity cDNA synthesis kit (Life Technologies). Gene expression levels were analyzed using TaqMan best coverage assay on demand (Life Technologies).

Akt phosphorylation and Seahorse analysis were done using a minimum of 5 cell culture replicates, whereas gene expression analysis was done on cell culture triplicates. The level of Akt phosphorylation was calculated as a percentage of the total amount of Akt and gene expression levels by the ddCt-method using the average of two reference genes for normalization. The Mito Cell Stress Test report generator from Seahorse Bioscience was used to calculate the basal and maximal respiration and spare capacity.
